# Unusual Causes of Lead Poisoning, with Report of a Case1Read at the 35th annual meeting of the Medical Association of Central New York, held at Syracuse, N. Y., October 21, 1902.

**Published:** 1903-01

**Authors:** John Zimmer

**Affiliations:** Rochester, N. Y.


### Unusual Causes of Lead Poisoning, with Report of a Case.[1]

By JOHN ZIMMER, M. D., Rochester, N. Y.

IN VIEW of the manifold and obscure channels through which lead may enter the system, as well as the difficulty oftentimes of referring the symptoms of intoxication to their proper source, especially when a knowledge of contact with the metal is to be acquired only by direct and searching inquiry, a brief consideration of this subject seems not without interest and importance, as lead, like the poor, we have with us always in some form or another.

Although almost every occupation produces a special liability to certain forms of disease, those who work in lead show by far the highest mortality, from an occupational standpoint. Manufacturers of white lead, painters, glaziers, plumbers, workers in rubber factories, and the like, are frequently the subjects of plumbism.

The danger of some of these occupations is so great, that states and countries have at different times attempted legislative control. Thus France in 1878, passed a law prohibiting the use of lead oxide in glazing pottery. In 1897, England also passed a similar law, after receiving the annual report of the chief inspector of factories, which showed that there had been 1,390 cases of lead poisoning, acute and chronic, among pottery workers, most of whom were women. As a result of this intoxication, abortion and sterility had become very common among them. In Philadelphia there is a local ordinance which prohibits the employment of laborers in the white lead factories for more than three months at a time.

It is not my purpose, however, in this paper to consider these common modes of infection, but rather some of the unusual causes of poisoning by this metal. A hurried review of the literature on this subject shows that considerable has been written along this line. Thus the Rome correspondent of the *London Lancet*, in August, 1896, reports a curious instance of wholesale lead poisoning, especially in Milan, caused in an unusual manner. It seems the sausage manufacturers paid the government tax on their products by means of a lead seal attached to the sausage. The sausage was cooked with the seal on, and the various salts contained in the meats, *e. g.*, nitrate of potash,

1. Read at the 35th annual meeting of the Medical Association of Central New York, held at Syracuse, N. Y., October 21, 1902.

chloride of sodium, and the like, together with the practice of washing the intestines with vinegar or sour wine, coming in contact with the lead oxide, all combined to produce a very active poison. The sausages are consumed in large quantities by the natives, and the liquor in which they have been boiled is distributed to the poor. It was only after colic had become epidemic, that an analysis of the sausages disclosed the cause. The lead seal has since been replaced by an innocuous metal.

Pel, of Amsterdam, in June, 1897, reports three cases of chronic plumbism. The first occurring in a shoemaker who was accustomed to hold tinned nails in his mouth while at work; the second was a cigar maker, using a board covered with lead plate, on which the cigars were cut with a knife, and then putting the knife in his mouth; the third was in a man employed in embedding diamonds in a warmed lead mass, manipulating this with his fingers moistened with saliva.

Ransome, in *British Medical Journal* of June, 1900, reports three cases of lead encephalopathy, caused by using diachylon ointment as an abortifacient. It seems the English druggists sell balls of this preparation, representing about 23 grams,for a half penny, which is used to a large extent among the poorer classes for the purpose of producing abortion. The practice has become so prevalent that steps have been taken to control the sale of this preparation.

Hahn, in *Archiv für Kinderheilkunde*, in August, 1900, reports two cases of convulsions in children resulting from the use of unguentum lead oxide for eczema of the face and head. Death occurred in one case, the diagnosis being made post-mortem.

Barnes, in *Archives of Pediatrics*, September, 1900, reports two cases of lead poisoning in children caused by the use of fire wood, consisting of staves of old barrels which had contained white lead, the fumes being inhaled and also entering the food. Convulsions was a marked feature of these cases. The diagnosis was made by the blue line at the gums and the finding of lead in the urine.

Cases have also been reported among the cutters of precious stones in the Jura mountains, who use a lead wheel for grinding. Hair dyes, face powders and lotions, are also occasional sources of this intoxication. A case in which paprika, adulterated with the red oxide of lead, was the means of infection, is reported in the *Medical Record* of January, 1898.

When lead has been taken into the system it becomes deposited in the various tissues or is discharged by the emunctories. It has been found in all of the tissues of the body. Ranon, a French observer, working experimentally with guinea pigs, found lead in the salivary and parotid glands, which explains the occasional parotiditis seen in man in cases of plumbism. It is eliminated mainly by the kidneys.

The symptoms of lead poisoning may be acute or chronic. The acute form is easily recognised, especially when there exists the characteristic colic and a history of contact with the metal. The chronic type, however, particularly where the symptoms are mild and there is no direct history of using lead, may cause some difficulty of recognition, as the following case suggests. The history is as follows:

W. S., age 29; male; married; U. S.; occupation, lens polisher. Family history negative. Personal history: had usual diseases of childhood. Three years ago was ill about ten days with what his physician called typhoid fever. Careful inquiry of the nature of this illness suggests the possibility of a mild plumbism, as the principal subjective symptoms were headache and vague pains throughout the body. Otherwise he has been perfectly well. Has worked at his occupation of lens polisher for past seven years.

*Present illness*—Patient presented himself at my office February 21, 1902, with the following clinical history: for the past ten days has had a gradual increasing headache, pain in back and legs, tingling and numbness of fingers, chills, sweating and unable to sleep.

*Present Condition*—Patient is well nourished, weighing 145 pounds; height, 5 feet 7 inches. Is a mouth-breather, owing to nasal obstruction caused by injury to nose when young. Pulse, 60; temperature, 98 2-5°; bowels regular; some anorexia; tongue clean; lungs, heart, kidneys, and the like, negative. Assuming the case one of myalgia, was treated with phenacetine and salol for two days with no result. February 24, patient complains of intense headache and backache, unable to rest or sleep. Pulse, 60; temperature, 98 2-5°. As he gave a history of having chills and sweating, quinine sulph. in large doses was prescribed and an examination of the blood made. The results were negative with no relief of pain. February 26, an examination showed some enlargement and tenderness of the liver; also considerable swelling and pain of right parotid gland. Pulse, 64; temperature, 101 2-5°. At this time there was seen to be a faint blue line on the gums. Plumbism was suspected, in spite of the fact that the patient was not aware of handling lead. A careful inquiry into the nature of his work showed that the lenses are polished by means of a paste or rouge on a soft buffing wheel. He was given a saturated solution of mag. sulp. until free catharsis occurred, and potass. iod., grs. v., 4xd. The next day patient felt some better and there was a continued improvement of all subjective symptoms from this date. No fever; pulse, 60. After giving potass. iodid. for five days, an examination of the urine by the potass. sulphide test showed the presence of lead. March 5, patient complained of cramps in calf muscles for the first time. These lasted three or four days, and then cleared up. The swelling of the liver and parotid gland also subsided. At this time it was noted that the pulse, which had always been in the neighborhood of 60, strong and full, now counted 90, at which point it remained some five or six days, and then subsided to normal. Lead was repeatedly found in the urine up to March 18, at which time the patient reported well and resumed his work. Later he procured a specimen of the rouge used in his work, and an analysis showed unmistakable evidence of lead, although not in large quantities. The base of the preparation is the red oxide of iron, so that the lead is probably an impurity.

Some interesting features of this case are, that although there are a great many polishers of lenses employed at this factory, the patient said that he had never heard of any of the other workmen being poisoned, although there were always a few sick with rheumatism, grippe, and the like. In the light of the above case it is possible that there may have been other cases which escaped recognition. Certainly it is not known among the workmen that they are handling lead.

Personal factors in this case might have been responsible for his susceptibility; as was previously stated the patient is inclined to breathe through his mouth, thus permitting the fine dust to pass more readily into the system. That the quantity of lead in the rouge is not large is suggested by the long term of constant employment at the work (seven years), and the rather vague and insidious onset of the intoxication, which indicates the gradual accumulation of small quantities, frequently and regularly repeated.

There was an entire absence of abdominal symptoms, *e. g*, constipation, colic and the like, but rather features suggesting inanition of the nerves and membranes of the brain, together with a low grade inflammation of the liver and parotid gland, which, had the process continued, would have lead to more serious changes, paralysis, wrist-drop, and the like.

In view of the obscure nature of this case it suggests that even where there is no direct history of working with lead, an examination of gums, urine and the like, might well be made a routine practice among workers in factories, where subjective symptoms are of an atypical character.

50 CUMBERLAND STREET.

### DISCUSSION.

Dr. C. E. CONGDON, Buffalo.—This paper has been extremely interesting to me, as it brings to mind that about ten years ago I was called by a general practitioner to see a case of appendicitis. The doctor thought the case was typhoid fever, or it had been diagnosticated so. This man already had passed through two hands, the diagnosis was appendicitis, and the medical treatment was being carried on. He suffered from diffuse pain in the abdomen; there had been no headache, no muscular pain; the history extended back about a year, with recurrent attacks of pain in the abdomen. Relief was obtained, he said, by small drinks of whiskey early in the morning. When I saw him the pulse was slow and full. The urine was not examined. I found no line upon the gum upon examination. He was quite tender in the abdomen, and there seemed to be considerable rigidity over a certain point. It looked very much like appendicitis, but that was not to be thought of, extending over that length of time, and I decided at once that he had lead poisoning. By the use of iodine of potassium, he was relieved in two or three days. It is well to bear in mind these conditions, and I think this paper is extremely timely, owing to the fact that lead poisoning can produce symptoms, abdominal manifestations, which will so closely simulate appendicitis.

Dr. ZIMMER.—I would like to add since writing this paper I have come across another case, in which a young man employed at cutting curtain shades showed unmistakable evidences of insidious lead-poisoning. His principal symptoms were malaise and vague pains, but not enough to keep him from his work. He looked anemic, and he did not know whether he was sick enough to come to a physician or not, but I examined his gums, and after giving him potassium iodide, I found lead in the urine. As I say, these cases are sometimes very obscure. Of course, this young man did not realise he was handling lead, although I have no doubt that the dust from cutting these curtain shades led to this affection.

---

WORMLEY (*Medical Age*) speaks of good results obtained in gastrointestinal catarrh by the use of cold compresses. They are applied as a girdle made of towels, wrung out of cold water and wrapped around the abdomen and left on all night. The relief from the flatulence and eructations, common in these patients, is remarkable.

